# Regulation of Mitochondrial Permeability Transition Pore Opening by Monovalent Cations in Liver Mitochondria

**DOI:** 10.3390/ijms24119237

**Published:** 2023-05-25

**Authors:** Ekaterina S. Kharechkina, Anna B. Nikiforova, Alexey G. Kruglov

**Affiliations:** Institute of Theoretical and Experimental Biophysics, Russian Academy of Sciences, Institutskaya 3, Pushchino, 142290 Moscow, Russia; katya.kypri@gmail.com (E.S.K.); nikiforanna@yandex.ru (A.B.N.)

**Keywords:** permeability transition pore, K^+^ channels, monovalent cations, phosphate carrier, K^+^/H^+^ exchanger

## Abstract

The opening of the permeability transition pore (PTP) in mitochondria is a key event in the initiation of cell death in various pathologic states, including ischemia/reperfusion. The activation of K^+^ transport into mitochondria protects cells from ischemia/reperfusion. However, the role of K^+^ transport in PTP regulation is unclear. Here, we studied the role of K^+^ and other monovalent cations in the regulation of the PTP opening in an in vitro model. The registration of the PTP opening, membrane potential, Ca^2+^-retention capacity, matrix pH, and K^+^ transport was performed using standard spectral and electrode techniques. We found that the presence of all cations tested in the medium (K^+^, Na^+^, choline^+^, and Li^+^) strongly stimulated the PTP opening compared with sucrose. Several possible reasons for this were examined: the effect of ionic strength, the influx of cations through selective and non-selective channels and exchangers, the suppression of Ca^2+^/H^+^ exchange, and the influx of anions. The data obtained indicate that the mechanism of PTP stimulation by cations includes the suppression of K^+^/H^+^ exchange and acidification of the matrix, which facilitates the influx of phosphate. Thus, the K^+^/H^+^ exchanger and the phosphate carrier together with selective K^+^ channels compose a PTP regulatory triad, which might operate in vivo.

## 1. Introduction

The opening of the PTP in mitochondria is a key event in the initiation of cell death and injury to organs and tissues in various pathological conditions, in particular, prolonged ischemia followed by reperfusion [1,2]. It is well known that the activation of K^+^ transport into mitochondria, e.g., using the activators of mitochondrial ATP-sensitive K^+^ (mK_ATP_) channels or Ca^2+^-activated K^+^ channels, has a significant protective effect in organs during ischemia/reperfusion (chemical pre- and post-conditioning) [3,4,5,6,7,8,9,10]. The genetic knockout of mitochondrial K^+^ channels and the application of their pharmacological inhibitors suppress the protective effect of both ischemic and chemical pre-conditioning [6,7,8,9,10]. However, the mechanism of the suppression of cell death upon activation of K^+^ channels is not completely understood. In particular, the role of the K^+^ transport in the induction of the PTP by Ca^2+^ is not clear. It was suggested that the activation of K^+^ transport causes mild mitochondrial uncoupling, which decreases the accumulation of Ca^2+^ by mitochondria and the generation of ROS and, thus, negatively regulates the PTP [11,12,13,14,15,16,17].

However, the data on the regulation of the PTP by modulation of K^+^ transport in isolated mitochondria deserve further clarification [4,5,16,17,18,19,20,21,22,23]. On the one hand, in the majority of studies, K^+^ fluxes were modulated by known activators and inhibitors of K^+^ channels, which have additional targets in mitochondria and exert effects on the PTP opening independent of K^+^ transport. For instance, the activator of mK_ATP_ channel diazoxide inhibits succinate dehydrogenase [24,25], while the inhibitors of the channel, 5-hydroxydecanoic acid and glibenclamide, can interact with mitochondrial ADP/ATP carriers (ANT) [26], important regulators of the PTP. Accordingly, the K^+^ transport-independent stimulation of PTP opening by glibenclamide was also reported [27]. On the other hand, the data on the effect of activation of K^+^ entry into mitochondria by low doses of the K^+^ ionophore valinomycin on Ca^2+^-retention capacity (CRC) (i.e., resistance to PTP opening in situ) are controversial [11,28]. Moreover, to our knowledge, the effect of the presence of K^+^ ions in the incubation medium on the PTP opening was not studied in detail previously.

**Figure 1:**
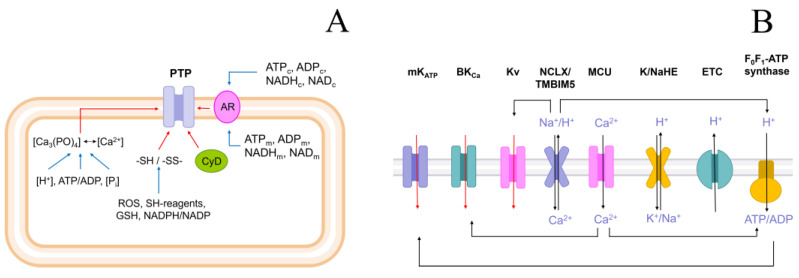
Regulation of PTP (**A**) and K^+^ channels in mitochondria (**B**). Designations: AR, allosteric regulators; CyD, Cyclophilin D; GSH, reduced glutathione; TMBIM5, Ca^2+^/H^+^ exchanger; mK_ATP_, ATP-sensitive K^+^ channel; BK_Ca_, large-conductance calcium-activated potassium channel; Kv, voltage-regulated potassium channel; NCLX, Na^+^/Ca^2+^/Li^+^ exchanger; MCU, mitochondrial Ca^2+^ uniporter; K/NaHE, K^+^/H^+^ exchanger/Na^+^/H^+^ exchanger; ETC, electron transport chain.

There are at least four classes of the regulators of PTP state, each of which can be affected by numerous modulators (Figure 1A). The first includes Ca^2+^ and some other divalent cations. In turn, the concentration of free Ca^2+^ in the mitochondrial matrix can be modulated by the matrix pH and the concentrations of inorganic phosphate (P_i_) and adenine nucleotides [29,30,31,32,33,34]. The second class comprises the vicinal thiol residues in several critical PTP regulators [35,36,37,38,39]. The redox state of critical thiols can be affected by GSH; ROS; and other free radicals, thiol reagents, and NADPH redox state [40,41,42,43,44,45,46]. Allosteric PTP regulators, including ATP/ADP-binding ANT and/or F-ATP synthase [47,48,49,50,51,52] and NAD(P)(H)-binding proteins of unknown nature make up the third class [53,54,55,56]. The fourth class includes the chaperone cyclophilin D (CyPD) [57,58,59]. The mitochondrial transmembrane potential (ΔΨ_m_) may be an additional PTP regulator since depolarization is known to stimulate PTP opening [41,60,61,62,63]. However, it is not clear whether the effect of ΔΨm on PTP is direct, or it involves other PTP regulators.

Theoretically, the activation of K^+^ entry into mitochondria can affect the state of at least three out of four regulators. Indeed, K^+^ entry can change the matrix pH due to the K^+^/H^+^ exchange [15,64,65,66]. It can alter the matrix ATP/ADP ratio due to the modulation of oxidative phosphorylation and anaerobic dephosphorylation [6,67]. Further, mild uncoupling caused by K^+^ entry may affect ROS production and NAD(P)H/NAD(P) ratio [12,13,67,68]. Finally, the activation of K^+^ entry can decrease the ΔΨ_m_ and the gating potential of the PTP [14,67]. The fact that several types of K^+^ channels have been found in mitochondria confirms the important role of K^+^ transport in mitochondrial physiology [69,70]. It is important that the majority of them may be directly or indirectly activated by Ca^2+^ (Figure 1B).

Therefore, the goal of this study was to examine the role of K^+^ in the regulation of the PTP in an in vitro model, to compare the effects of K^+^ and other monovalent cations, and to elucidate the mechanisms of this regulation.

## 2. Results

### 2.1. Effect of K^+^ and Na^+^ in the Incubation Medium on the Rate of PTP Opening

**Figure 2:**
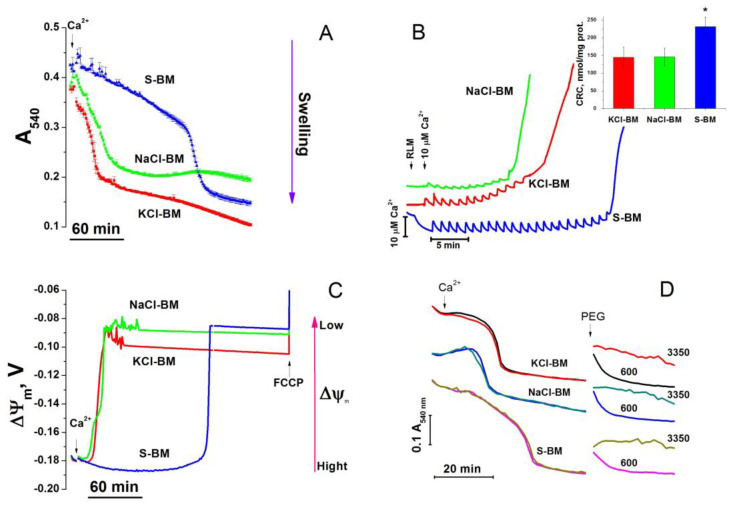
Ca^2+^-dependent PTP opening in mitochondria incubated in KCl-, NaCl-, and S-BM: swelling (**A**), CRC (**B**), dissipation of ΔΨ_m_ (**C**), and PEG-dependent shrinkage of swollen mitochondria (**D**). Two minutes before measurements, mitochondria (0.75 (**A**,**C**,**D**) or 1 mg protein/mL (**B**)) were placed in the incubation media supplemented with 5 mM glutamate, 5 mM malate, and 10 µM EGTA (**A**,**C**,**D**). The media also contained 330 nM rhodamine 123 (**C**). (**A,C**) Five minutes after the beginning of recordings, 20 µM CaCl_2_ (final concentration) was added to initiate PTP opening. Standard traces of one representative parallel experiment of three that are similar are presented. Values on traces are means (±S.D.) for three technical replicas. (**B**) Pulses of Ca^2+^ (10 nmol/mg protein) were added each 1.5 min. Traces in KCl- and NaCl-BM were shifted up by 5 and 15 µM, respectively, for visual separation. The insert shows the mean CRC values ± S.E.M. (*n* = 11) of four independent experiments. An asterisk indicates significant difference between S- and saline-based media (*p* < 0.01). (**D**) Arrows show the addition of 10 (KCl-/NaCl-BM) or 30 µM Ca^2+^ (S-BM) and 600 or 3350-Da PEGs. Curves recorded in different media were shifted along the *Y*-axis for visual separation. Values in traces are the means of three technical replicates.

Previously, it was shown that in isolated mitochondria in the absence of added ATP, mK_ATP_ channels are opened [65]. The addition of Ca^2+^ to the mitochondria should lead to an opening of Ca^2+^-activated and voltage-gated K^+^ channels, the latter due to transient membrane depolarization [71]. It is logical to suggest that if K^+^ inward transport through any channel type is essential for mitochondrial protection, organelles will be more resistant to Ca^2+^-induced PTP opening in the K^+^-containing medium. Figure 2 demonstrates the kinetics of Ca^2+^-dependent high-amplitude mitochondrial swelling (A) and the dissipation of ΔΨ_m_ (C), as well as the results of the measurements of CRC (B) in the mitochondria incubated in KCl-based medium (KCl-BM), NaCl-based medium (NaCl-BM), and sucrose-based medium (S-BM). Recordings in all media were started after approximately 2-min of incubation of the mitochondria with the respiratory substrate (5 mM glutamate, 5 mM malate) for the initial equilibration of ion and solute concentrations. As follows from the figure, the longest time between the addition of Ca^2+^ (20 µM, 26.6 nmol/mg protein) and the initiation of mitochondrial swelling (A) and the dissipation of ΔΨ_m_ (C) was observed in S-BM. In KCl- and NaCl-BM, the swelling and ΔΨ_m_ loss were initiated four to eight times faster, indicating a lower resistance of the mitochondria to PTP opening in cation-containing media.

Mitochondrial swelling and the dissipation of ΔΨ_m_ can be due to both PTP opening and the electrophoretic influx of cations through the activated cation channels (regardless of their selectivity). To examine the latter possibility, we added 600- and 3350-Da polyethylene glycols (PEGs) to swollen mitochondria and observed either transient or sustainable mitochondrial shrinkage, respectively. These data clearly indicate that the inner mitochondrial membrane (IMM) contains opened pores permeable for solutes of molecular weight higher than 600 but lower than 3350 Da, i.e., the PTP. In accordance with a slower PTP opening, the highest CRC (B) was observed in S-BM (232 ± 26 vs. 144.7 ± 28.6 (KCl-BM) and 146 ± 24.4 nmol∙min^−1^∙mg prot^−1^ (NaCl-BM)). Thus, the presence of K^+^ or Na^+^ ions in the incubation medium did not postpone but considerably stimulated the PTP opening.

There are several possible reasons for the stimulation of PTP opening in the presence of salts: the effect of the ionic strength, the triggering of the PTP opening by the influx of solutes into the matrix, and the effect of the salts on the PTP regulation independently of the influx of cations or anions. All of these reasons were experimentally examined.

### 2.2. Effect of the Concentration of Salts in the Incubation Medium on the Rate of PTP Opening

**Figure 3:**
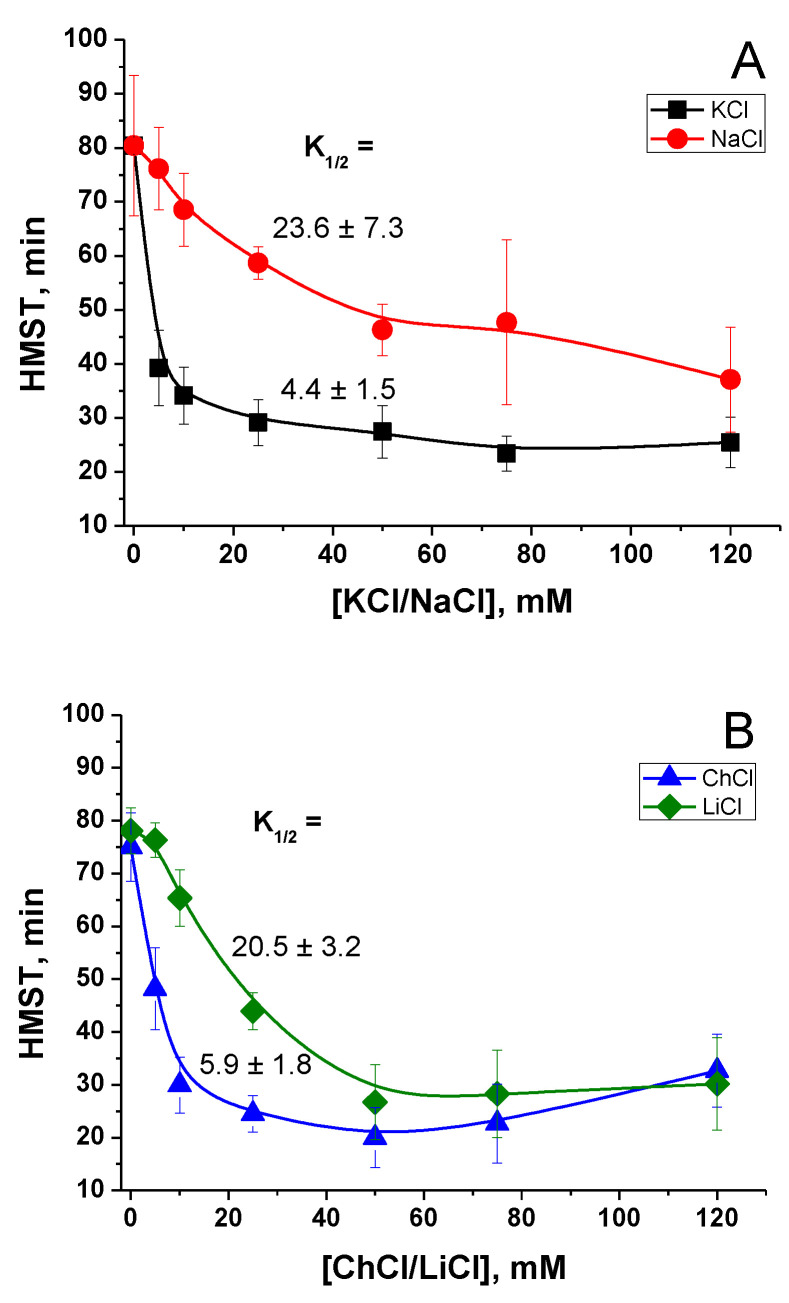
Effect of KCl, NaCl, ChCl, and LiCl concentration in the incubation medium on the rate of PTP opening. Mitochondria (0.75 mg protein/mL) were placed in the basic sucrose/HEPES/H_3_PO_4_/MgCl_2_ media supplemented with salts at indicated concentrations, respiratory substrates glutamate and malate (5 plus 5 mM), sucrose to equilibrate the osmolarity (295 mOsM), and 10 µM EGTA. Five minutes after the beginning of the recordings, 20 µM CaCl_2_ (final concentration) was added to initiate PTP opening. HMST values were determined as described in Materials and Methods. (**A**) Titration of HMST with KCl and NaCl. (**B**) Titration of HMST with ChCl and LiCl. Values on traces are the means ± S.E.M. of five (**A**) and three (**B**) independent titrations. Numbers at traces are the means ± S.E.M. of K_1/2_ values (concentrations of salts required to reduce the protective effect of sucrose by half) in mM.

The ionic strength of media may affect the interaction of charged groups of proteins of a PTP complex and membrane phospholipids, thus stimulating the pore opening. We measured the rate of PTP opening in the media containing different concentrations of KCl, NaCl, LiCl, and choline-Cl (ChCl) in S-BM (Figure 3). The final osmolarity of all media was about 300 mOsM, while the ionic strength varied from ~15 mM (S-BM) to 135 mM (maximum concentration of salts). The rate of PTP opening in a mitochondrial suspension was defined as the time required for half-maximal swelling (HMST).

Figure 3 shows that the concentrations of KCl and ChCl that reduced the protective effect of S-BM by half (K_1/2_) were as low as 4.4 ± 1.5 and 5.9 ± 1.8 mM, respectively. The concentrations of LiCl and NaCl required for a 50% reduction of sucrose protection were somewhat higher at 20.5 ± 3.2 and 23.6 ± 7.3 mM, respectively. Thus, the PTP opening can be triggered by various cations. Nevertheless, K^+^ and Ch^+^ are three to five times more efficient as triggers than Na^+^ and Li^+^. The low concentrations of salts required for PTP stimulation count rather in favor of mechanisms involving ion channels and exchangers. Moreover, the fact that an increase in ionic strength from 15 to 19–20 mM reduces the protection by a factor of two (KCl and ChCl) implies the existence of too sharp a threshold in the dependence of ionic interactions on the ionic strength of the medium. Thus, these data show that the contribution of the ionic strength to the stimulation of PTP opening is very unlikely. 

Then, we explored whether the stimulation of PTP opening in the presence of salts is a consequence of the electrophoretic influx of cations via any mechanism.

### 2.3. Role of K+ Channels in the Stimulation of PTP Opening

**Figure 4:**
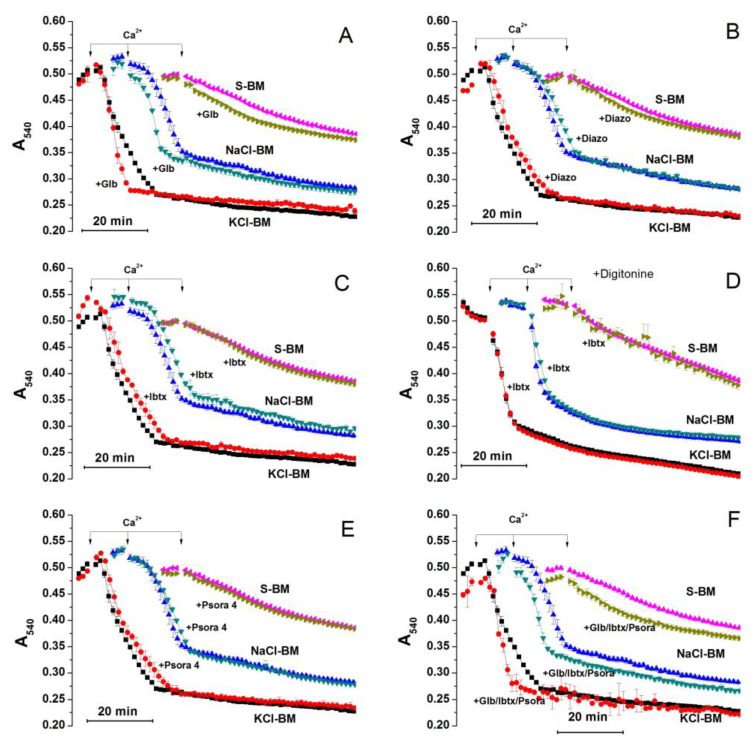
Effect of modulators of mitochondrial K^+^ channels on the kinetics of PTP-related mitochondrial swelling in KCl-, NaCl-, and S-BM. Two minutes before measurements, mitochondria (0.75 mg protein/mL) were placed in incubation media supplemented with 5 mM glutamate plus 5 mM malate (Trizma salts), 10 µM EGTA and, where indicated, 5 µM glibenclamide (**A**,**F**, +Glb), 30 µM diazoxide (**B**, +Diazo), 100 nM iberiotoxin (**C**,**D**,**F**, +Ibtx), 30 nM psora 4 (**E**,**F**), and 100 µM digitonin (**D**). Four (all panels except D) or seven (**D**) minutes after the beginning of simultaneous measurements in three media, 20 µM CaCl_2_ (final concentration) was added to initiate PTP opening. In panels, traces were shifted by 10 (NaCl-BM) and 25 min (S-BM) for clarity. Arrows show the moment of Ca^2+^ addition. Traces of two experiments of four similar are presented. The points of traces are the means ± S.E.M. of three technical replicates.

Potassium was the strongest PTP trigger among the cations tested. Thus, we examined the effect of potent and selective modulators of K^+^ channels on the PTP opening in KCl-, NaCl-, and S-BM. Figure 4 demonstrates the effect of glibenclamide (A), diazoxide (B) (an inhibitor (K_1/2_ = 0.04–5 μM), and an activator of mK_ATP_ channel (K_1/2_ = 0.5–0.8 μM), respectively [7,72]); the effects of iberiotoxin (C and D) (an inhibitor of BK_Ca_ channel, K_1/2_ ≤ 100 nM [3]), psora 4 (E) (an inhibitor of Kv_1.3_ channel, K_1/2_ = 3 nM [73]), and of a combination of the inhibitors (F) are also shown. As psora 4 can also inhibit Kv_1.1_, Kv_1.2_, Kv_1.4_, and Kv_1.7_ channels, though with lower efficiency, we assessed its effect in the 30–1000-nM range in separate experiments (Supplementary Material Figure S1.) Since a 4-kDa iberiotoxin molecule may be too large to pass through the VDAC channels freely, we examined its effect in mitochondria with both an intact outer membrane (C) and a membrane damaged by 100 µM digitonin [74]. As can be seen, the effect of all modulators and the combination of inhibitors tested were either weak or unspecific for K^+^ or both (A–F). Thus, the entry of cations into the mitochondria through selective K^+^ channels seems to have minimum influence on PTP induction.

### 2.4. Role of Cations in the Transition of PTP from a Low- to a High-Conductance State

**Figure 5:**
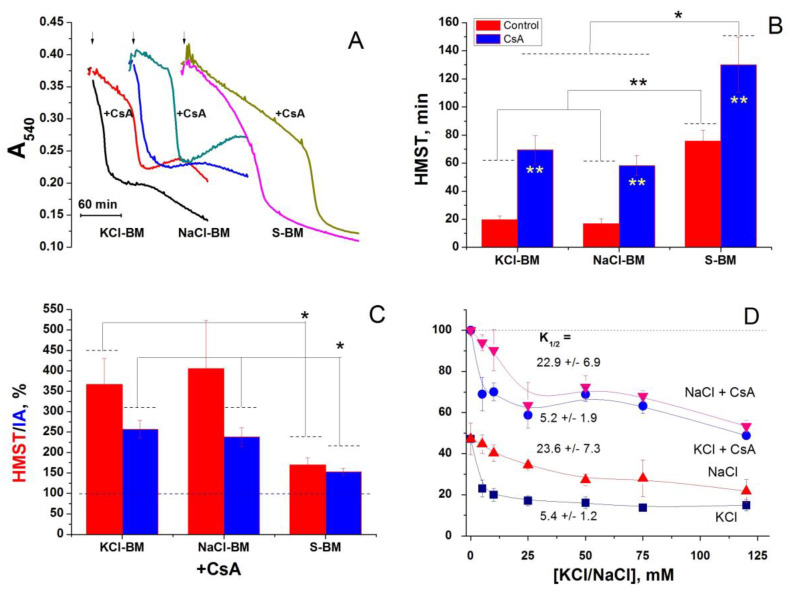
Effect of cyclosporin A on the rate of PTP opening in the cation-free (S-BM) and cation-containing media (KCl-BM and NaCl-BM). Mitochondria (0.75 mg/mL) were placed in the media supplemented with 5 mM glutamate, 5 mM malate, 10 µM EGTA and, where shown, 1 μM CsA. After a 5-min incubation, 20 µM Ca^2+^ was added. (**A**). Standard curves of mitochondrial swelling in KCl-, NaCl-, and S-BM in the absence and presence of CsA. Values on the curves are the means of three technical replicates. (**B**,**C**). Mitochondrial HMST and relative HMST and IA calculated for experimental conditions presented in panel A. HMST/IA in the absence of CsA correspond to 100% (dashed line). Values in columns are the means ± S.E.M. of at least three independent experiments. In all media, the difference between CsA-containing samples and controls is significant (*p* < 0.01). Asterisks show the statistically significant difference: *p* < 0.05 (*), *p* < 0.01 (**). (**D**). Dependence of the relative HMST on the concentration of KCl and NaCl in the incubation media in the presence and absence of CsA. HMST in S-BM in the presence of CsA was taken as 100% and in this experimental series corresponds to 170.5 ± 10.4 min. Figures at the curves indicate the salt concentrations required for the 50% suppression of the protective effect of sucrose (K_1/2_). The points of the traces are the means ± S.E.M. of four independent experiments. Arrows show the addition of 20 µM Ca^2+^ (final concentrations).

PTP can be in different states of channel conductance and transit from one state to another [28,74,75,76,77,78]. Cyclosporin A (CsA), an inhibitor of CyPD, can constrain both full-conductance PTP opening and transitions between states [74]. We hypothesized that Ca^2+^ initially transforms the PTP into a K^+^/Na^+^-channel and stimulates the inward cation flow, which triggers full PTP opening. If this is true, CsA should decrease the efficiency of PTP triggering by cations. Therefore, we studied the effect of 1 µM CsA on the Ca^2+^-dependent swelling in K^+^/Na^+^-containing media and S-BM (Figure 5). As expected, CsA strongly inhibited Ca^2+^-dependent swelling both in K^+^/Na^+^-containing and cation-free media (Figure 5A), indicating the suppression of the PTP opening. The relative potency of the CsA used to protect the mitochondria was more pronounced in cation-containing media (~300% vs. ~180%) (Figure 5B), which could support our hypothesis. However, the absolute increment in HMST and integrated amplitude of absorbance (IA) was similar in all media (Figure 5C). The presence of CsA in the incubation media had a minor effect on the relative capability of KCl and NaCl to stimulate the PTP opening: the K_1/2_ for the salts was 5.2 ± 1.9 and 22.9 ± 6.9 mM, respectively (Figure 5D). These things considered, CsA was almost equally efficient in protecting the mitochondria in the media of different salt concentrations. These data indicate that the removal of cations and the suppression of CyPD additively contribute to PTP inhibition.

### 2.5. Role of Non-Selective Cation Channel in the PTP Triggering by Monovalent Cations

**Figure 6:**
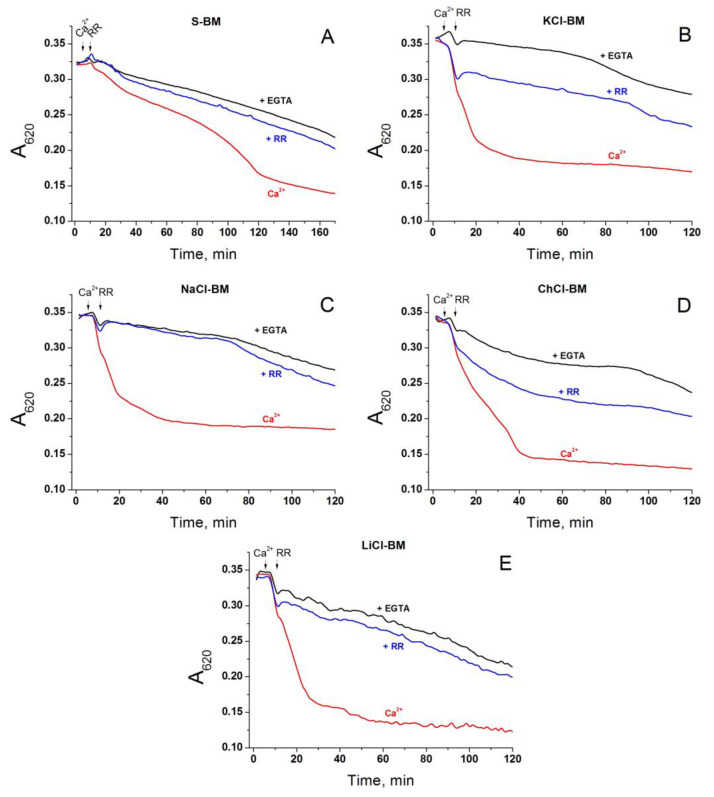
Effect of RR on the rate of PTP opening in Ca^2+^-loaded mitochondria in S- (**A**), KCl- (**B**), NaCl- (**C**), ChCl- (**D**), and LiCl-based medium (**E**). Two minutes before measurements, mitochondria (0.75 mg protein/mL) were placed in the corresponding incubation media supplemented with 5 mM glutamate plus 5 mM malate (Trizma salts) and 10 µM EGTA. Just before measurements, the suspensions were placed in the wells of 96-well plates, which, where shown, contained 1 mM EGTA (+EGTA). Arrows indicate the addition of 20 µM Ca^2+^ (final concentrations) and 1 µM RR. Representative traces of one experiment of three that are similar are presented. The points of the curves are the means of three technical replicates.

One more way for the electrophoretic entry of cations into the matrix of energized mitochondria to trigger the PTP opening is the hypothetical ruthenium red (RR)- and Mg^2+^-sensitive non-selective cation channel [79]. Though the non-selective cation channel is thought to be inhibited by submicromolar Mg^2+^ from both sides of the IMM, one may assume that the accumulation of Ca^2+^ abolishes the inhibition. If this assumption is correct, the addition of RR after the total accumulation of Ca^2+^ via the mitochondria will inhibit the PTP opening in the cation-containing media but not in S-BM. Figure 6 shows that 1 µM RR being added after Ca^2+^ strongly inhibited PTP opening both in cation-based media and S-BM. Since RR is a potent inhibitor of mitochondrial Ca^2+^ uniporter (MCU) and can prevent Ca^2+^ cycling between cytosolic and matrix sides of the IMM via the MCU and the Ca^2+^/H^+^ antiporter (TMBIM5), the data obtained are in favor of this mechanism but not the mechanism involving the non-selective cation channel.

**Figure 7:**
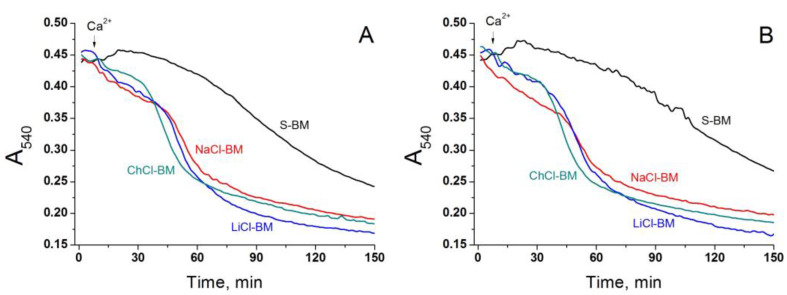
Effect of A23187 and RR on Ca^2+^-dependent swelling of de-energized mitochondrial in different media. Two minutes before measurements, mitochondria (0.75 mg protein/mL) were placed in the corresponding incubation media supplemented with rotenone (2 µg/mg), antimycin A (2 µg/mg), 1 µM myxothiazol, 500 nM FCCP, 10 µM A23187, 10 µM EGTA (**A**,**B**), and 1 µM RR (**B**). Then, the suspensions were placed in the wells of 96-well plates for measurements. Arrows indicate the addition of 50 µM Ca^2+^ (final concentrations). Representative traces of one experiment of three that are similar are presented. The points of the curves are the means of three technical replicates.

In order to further explore the role of the non-selective cation channel in the PTP triggering, we applied a model of de-energized mitochondria supplemented with Ca^2+^- and Mg^2+^-ionophore A23187, which bypasses the MCU. In these conditions, the entry of monovalent cations should be suppressed, which, in turn, must eliminate the difference in the swelling rate between mitochondria in S-BM and salt-based media. As follows from Figure 7, this is not the case. Mitochondria in the salt-based media (NaCl-, ChCl-, and LiCl-BM) swelled two times faster than those in S-BM (Figure 7A). However, the swelling was unaffected by RR (Figure 7B). Thus, the entry of Ca^2+^ into the matrix through bypassing the MCU suppresses the ability of RR to inhibit mitochondrial swelling. One can conclude that the hypothetic non-selective cation channel does not contribute to the PTP triggering by monovalent cations.

### 2.6. Role of IMAC in the Acceleration of PTP Opening in Salt-Containing Media

**Figure 8:**
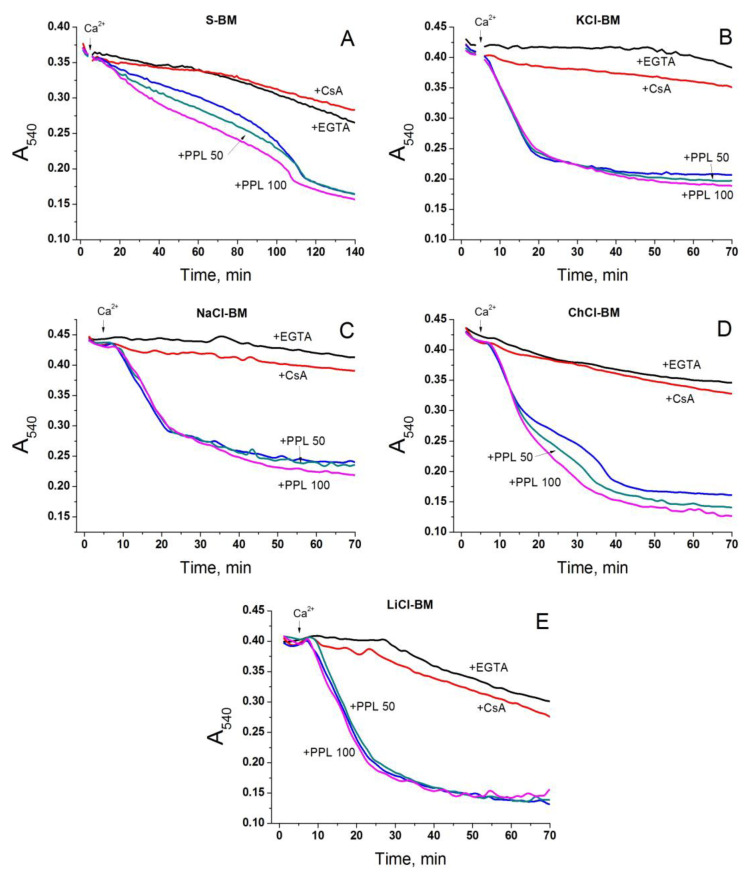
Effect of propranolol on the Ca^2+^-dependent mitochondrial swelling in KCl-, NaCl-, ChCl-, LiCl-, and S-BM. Two minutes before measurements, mitochondria (0.75 mg protein/mL) were placed in the incubation media supplemented with 5 mM glutamate plus 5 mM malate (Trizma salts), 10 µM EGTA and, where indicated, 1 mM EGTA (+EGTA), 1 µM CsA, and 50 and 100 µM propranolol (+PPL). Five minutes after the start of the recordings, 20 µM Ca^2+^ was added to the samples with standard (10 µM) EGTA. Incubation media are S-BM (**A**), KCl-BM (**B**), NaCl-BM (**C**), ChCl-BM (**D**), and LiCl-BM (**E**). Traces of one representative experiment of three that are similar are presented. The points of the curves are the means of three technical replicates. Arrows show the addition of 20 µM Ca^2+^ (final concentrations).

One may suggest that PTP opening is triggered by chloride anions, not cations. In order to address this possibility, we studied the effect of potent inhibitors of the Inner Membrane Anion Channel (IMAC), namely propranolol (IC_50_ ~ 25 µM) and Ro5-4864 (IC_50_ ~34 µM), on the Ca^2+^-dependent swelling in different media (Figure 8, Supplementary Material Figure S2) [80]. Propranolol (50 and 100 µM) (+PPL) clearly stimulated PTP-dependent swelling in S- and ChCl-BM (A and D) but had a minimal effect when in KCl-, NaCl-, and LiCl-BM (B, C, and E). Ro5-4864 (Ro) in 75 and 150 µM concentrations caused a dose-dependent stimulation of the swelling, even before the Ca^2+^ addition to all media tested (Supplementary Material Figure S2). These data do not confirm the importance of the entry of anions into the matrix through the IMAC for the stimulation of PTP opening. The activation of swelling may reflect the unspecific effects of the inhibitors on the system of volume regulation.

A mechanism underlying the higher susceptibility of the mitochondria to PTP opening in cation-containing media may involve exchanger-mediated ionic transport. Mitochondria contain systems capable of transporting K^+^, Na^+^, Li^+^, Ch^+^, and Ca^2+^ ions across the IMM in exchange for H^+^, namely K^+^ (Na^+^, Li^+^)/H^+^ exchanger (unidentified, presumably Letm1) [81], choline transporter-like protein 1(CTL1)/SLC44a1 [82], and Ca^2+^/H^+^ antiporter (TMBIM5) [83]. Moreover, the tissue-specific Ca^2+^/Na^+^ exchanger (SLC8b1/NCLX) is important for mitochondrial Ca^2+^ homeostasis [84]. It was earlier shown that the K^+^/H^+^ exchanger can regulate PTP opening in de-energized liver mitochondria [85]. Therefore, we explored whether the cation influx mediated by some cation/H^+^ exchangers accelerates the PTP opening in energized mitochondria.

### 2.7. Effect of the Inhibition of K^+^(Na^+^, Li^+^)/H^+^ and Ch^+^/H^+^ Exchangers on the PTP Opening in Different Media

**Figure 9:**
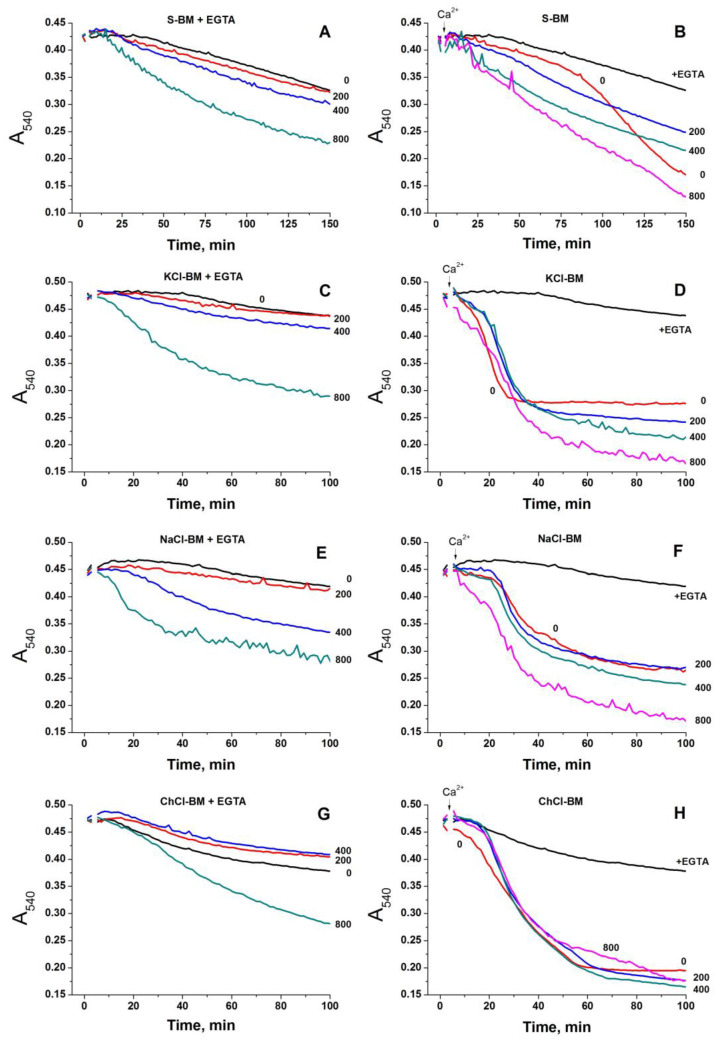
Effect of quinidine on Ca^2+^-dependent and -independent mitochondrial swelling in KCl-, NaCl-, ChCl- and S-BM. Two minutes before measurements, mitochondria (0.75 mg protein/mL) were placed in the incubation media supplemented with 5 mM glutamate plus 5 mM malate (Trizma salts), 10 µM EGTA, and indicated concentrations (µM) of quinidine. Where shown, the media contained 1 mM EGTA (+EGTA) instead of the standard 10 µM concentration. Five minutes after the start of recordings, 20 µM Ca^2+^ was added to the samples with standard EGTA. Incubation media are S-BM (**A**,**B**), KCl-BM (**C**,**D**), NaCl-BM (**E**,**F**), and ChCl-BM (**G**,**H**). Traces of one representative experiment of three that are similar are presented. The points of the curves are the means of three technical replicates. Arrows show the addition of 20 µM Ca^2+^ (final concentrations).

The activity of the K^+^(Na^+^, Li^+^)/H^+^ exchanger is sensitive to Mg^2+^, quinine, and its enantiomer quinidine. Both quinine and quinidine at a concentration of 0.5 mM cause almost complete inhibition of the carrier [64,86,87]. In addition, CTL1/SLC44a1 is sensitive to quinine and quinidine (K_i_~33–40 µM) [82,88,89]. Since the Mg^2+^ present in the cytosol in near-millimolar concentrations is an important modulator of the PTP, we evaluated the effect of the inhibitors of the K^+^(Na^+^, Li^+^)/H^+^ exchanger in intact energized mitochondria in different media supplemented with Mg^2+^. The rate of Ca^2+^-dependent (PTP) and -independent swelling was titrated by increasing the concentration of quinidine (Figure 9). 

In all media tested (S-, KCl-, and NaCl-BM), except ChCl-BM, quinidine dose-dependently stimulated PTP-independent swelling in the presence of EGTA (Figure 9A,C,E,G). This implies that the inhibitor shifted the balance of the entry and exit of the ions and solutes (K^+^, Na^+^, Pi, glutamate, malate) to netto entry. In NaCl-BM, quinidine was a more potent swelling inducer than in other media. In ChCl-BM, the inhibitor at low concentrations was rather protective, indicating that Ch^+^ is transported to the matrix almost exclusively via the quinidine-sensitive Ch^+^/H^+^ exchanger [88,89]. Quinidine at concentrations higher than 0.5 mM seems to cause unspecific mitochondrial damage. By contrast, Ca^2+^-dependent swelling (PTP) was relatively weakly modulated by quinidine in all cation-containing media (Figure 9D,F,H). In NaCl-BM, the effect was minimal. In ChCl- and KCl-BM, quinidine slightly postponed the onset of swelling. In addition, in KCl-BM, the amplitude of swelling increased as a function of quinidine concentration.

In S-BM, the effect of the suppression of the K^+^/H^+^ exchanger on Ca^2+^-dependent swelling was two-fold: an increase in the initial swelling but a decrease in the total swelling amplitude in long-term experiments due to the elimination of visible cooperativity (B). A reason for this effect will be revealed in the following sections. Nevertheless, the data obtained clearly demonstrate that cations do not trigger PTP opening via entry through the K^+^(Na^+^, Li^+^)/H^+^ and Ch^+^/H^+^ exchangers. 

These things considered, we checked the effect of the selective inhibitor of the Ca^2+^/Na^+^ exchanger (SLC8b1/NCLX) CGP-37157 on PTP opening in different media. As the carrier is abundant in heart mitochondria but absent in liver mitochondria, the effect of its inhibitor was expectedly negligible (Supplementary Figure S3).

### 2.8. The Rate of Ca^2+^ Extrusion from Mitochondria in Different Incubation Media

**Figure 10:**
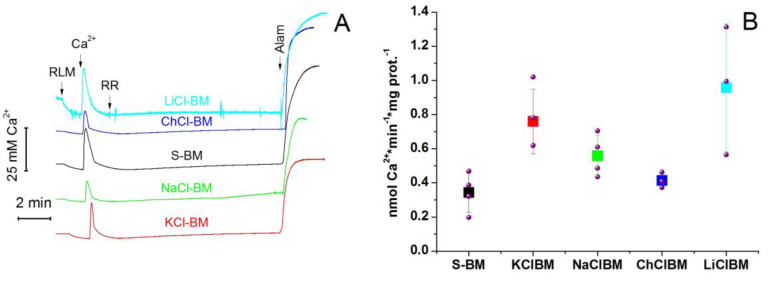
The rate of Ca^2+^ efflux from mitochondria in different incubation media. (**A**). Incubation media contained 5 mM glutamate plus 5 mM malate (Trizma salts), 1 µM CsA, and 10 µM EGTA. Arrows show the additions of mitochondria (1 mg protein/mL), 50 µM Ca^2+^, 1 µM RR, and 40 µg/mL Alam. Curves recorded in different media were shifted along the *Y*-axis for visual clarity. Standard traces of one representative experiment of three or four similar are presented. (**B**). The rates of Ca^2+^ efflux are presented as means ± S.E.M (for KCl-BM, NaCl-BM, S-BM *n* = 4, for ChCl-BM and LiCl-BM *n* = 3).

The inhibition of PTP opening by RR is most likely due to the Ca^2+^ extrusion via the Ca^2+^/H^+^ (cation) exchanger(s) when the Ca^2+^ influx via MCU is blocked (Figure 6 and Figure 7). One could expect that the rate of Ca^2+^ extrusion should inversely correlate with the rate of PTP opening. In order to address this issue, we measured the rate of the release of accumulated Ca^2+^ by mitochondria treated with RR in different incubation media (Figure 10). The release of Ca^2+^ was measured using a Ca^2+^-selective electrode. The media contained 1 µM CsA in order to inhibit PTP opening. At the end of each recording, alamethicin (Alam) was added to the medium to assess the total amount of Ca^2+^ accumulated by mitochondria. Figure 10 shows that the presence of salts in the medium did not reduce the rate of Ca^2+^ extrusion from mitochondria. On the contrary, the minimum rate of Ca^2+^ release was observed in S-BM. In ChCl-BM it was almost the same as in S-BM. In other media, it was approximately 2.0 (KCl-BM), 1.6 (NaCl-BM), and 2.7 times higher (LiCl-BM) than in S-BM. Thus, the rate of Ca^2+^ release in various media is not the main factor that determines the sensitivity of mitochondria to PTP induction in the presence of salts.

### 2.9. Effect of External pH on PTP Opening in Different Media

**Figure 11:**
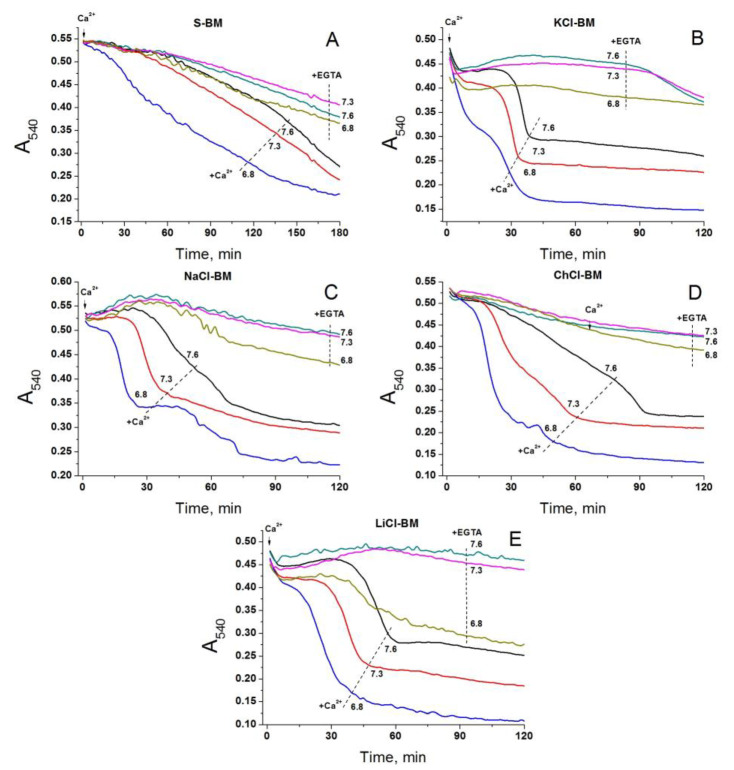
Effect of the pH of S- (**A**), KCl- (**B**), NaCl- (**C**), ChCl- (**D**), and LiCl-BM (**E**) on the rate of Ca^2+^-dependent PTP opening. Two minutes before measurements, mitochondria (0.75 mg protein/mL) were placed in the corresponding incubation media (pH 7.6) supplemented with 5 mM glutamate plus 5 mM malate (Trizma salts) and 10 µM EGTA. Just before measurements, the suspensions were placed in the wells of 96-well plates, which, where shown, contained 1 mM EGTA (+EGTA), 20 µM Ca^2+^ (final concentrations), and 1.05 (pH 7.3) or 2.4 µL/100 µL (pH 6.8) of 400 mM HCl. Figures at traces show the pH of the media. Representative traces of two experiments of four that are similar are presented. The points of the curves are the means of three technical replicates. Arrows show the addition of 20 µM Ca^2+^ (final concentrations).

The pH of incubation media is an efficient regulator of the activity of all H^+^/OH^−^-dependent symporters and antiporters [83,90,91,92]. More acidic external pH stimulates the extrusion of cations and entry of anions, e.g., of P_i_ and glutamate. Therefore, if exchanger-mediated cation entry into the matrix is essential for the triggering of the PTP, the latter will open more readily at an alkaline external pH. Conversely, if the PTP opening is activated by the entry of anions, the acidic external pH will stimulate the pore opening. The data presented in Figure 11 shows that in all media tested, namely S- (A), KCl- (B), NaCl- (C), ChCl- (D), and LiCl-BM (E), the Ca^2+^-dependent PTP opening occurred most rapidly at pH 6.8 and most slowly at pH 7.6. Hence, conditions that hamper the cation entry through any H^+^-dependent antiporter but promote the acidification of the matrix via the antiporters, or P_i_ and glutamate carriers, strongly stimulate PTP opening [92]. Further, at all the pH levels tested, the swelling was faster in cation-containing media than in sucrose. One can conclude that the decrease in the matrix pH but not the exchanger-dependent entry of cations underlies the effect of external pH on the rate of PTP opening.

### 2.10. Dynamics of Matrix pH in Different Incubation Media

**Figure 12:**
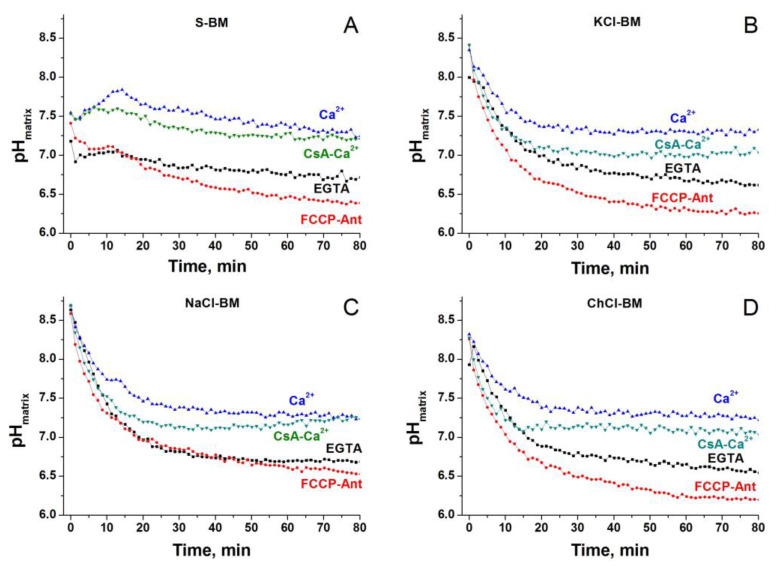
Dynamics of matrix pH in different media. Two minutes before measurements, Mitochondria (0.75 mg protein/mL) pre-loaded with 2′,7′-bis(carboxyethyl)-5,6-carboxyfluorescein (BCECF-AM) were placed in the corresponding incubation media supplemented with 5 mM glutamate plus 5 mM malate (Trizma salts) and 10 µM EGTA. Just before measurements, the suspensions were placed in the wells of 96-well plates, which, where shown, contained 1 mM EGTA, 20 µM Ca^2+^, 1 µM CsA, 500nM FCCP, and antimycin A (Ant) (2 µg/mg). Representative traces of one experiment of three that are similar are presented. The points of the curves are the means of three technical replicates. Incubation media are S-BM (**A**), KCl-BM (**B**), NaCl-BM (**C**), and ChCl-BM (**D**).

The pH of the mitochondrial matrix is a strong regulator of the PTP, which can modulate the degree of protonation of critical amino acid residues that regulate the PTP [93], the stability of Ca^2+^-P_i_ complexes [30], and the rate of transport of P_i_ and substrates. We assumed that cations in the media change the matrix pH in a way of facilitating the PTP opening. Therefore, we measured matrix pH in mitochondria incubated in S-, KCl-, NaCl-, and ChCl-BM in the presence and absence of Ca^2+^, the inhibitor of the respiratory chain antimycin, and an uncoupler (Figure 12). 

Approximately in the first 20 min of incubation in the absence of Ca^2+^, the matrix pH in S-BM was much lower than in the salt-based media (~7.0 versus 8.0–8.7). The addition of Ca^2+^ to the media caused the alkalization of the matrix in S-BM and suppressed acidification in saline media. In the presence of CsA, the Ca^2+^ effect was less pronounced. In all media, the lowest pH of the matrix was observed in the presence of antimycin and the uncoupler. 

The effect of the addition of A23187 to energized mitochondria confirms these observations (Supplementary Material Figure S4). In S-BM, A23187 increased Ca^2+^-dependent swelling, which indicates its operation in the mode of Ca^2+^ injection in exchange for the matrix H^+^. In the presence of salts, A23187 inhibited PTP opening because a more alkaline matrix pH facilitates the extrusion of Ca^2+^ in exchange for external H^+^.

### 2.11. Cations Regulate the Activity of K^+^/H^+^ Exchanger, Matrix pH, and P_i_ Influx

**Figure 13:**
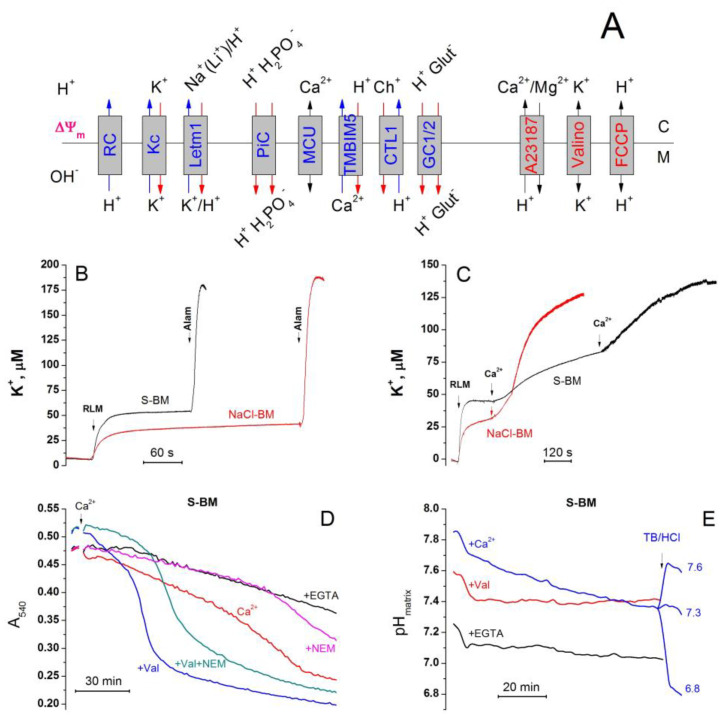
Cations regulate the activity of the K^+^/H^+^ exchanger, matrix pH, and P_i_ influx. (**A**). Transporters, exchangers, and ionophores capable of regulating cation and anion fluxes and matrix pH. Designations: RC, respiratory chain; Kc, K^+^ channel; Letm1, K^+^(Na^+^, Li^+^)/H^+^ exchanger; TMBIM5, Ca^2+^/H^+^ exchanger; CTL1, choline transporter-like protein 1; GC1/2, glutamate carriers 1 and 2; Valino, valinomycin. B and C. K^+^ efflux from mitochondria in NaCl-BM and S-BM. Incubation media contained 5 mM glutamate plus 5 mM malate (Trizma salts) and 10 µM EGTA. Arrows show the additions of RLM (1 mg protein/mL), 40 µg/mL Alam (**B**), and 50 µM Ca^2+^ (**C**). Standard traces of one representative experiment of three that are similar are presented. (**D**,**E**) The impact of valinomycin and N-ethylmaleimide on PTP opening and matrix pH of RLM in S-BM. Two minutes before measurements, mitochondria (0.75 mg protein/mL) (**D**) and mitochondria (0.75 mg protein/mL) pre-loaded with BCECF-AM € were placed in the S-BM supplemented with 5 mM glutamate plus 5 mM malate (Trizma salts), and 10 µM EGTA. Just before measurements, the suspensions were placed in the wells of 96-well plates which, where shown, contained 1 mM EGTA (+EGTA), 50 ng/mL valinomycin (+Val), 10 μM NEM (+NEM), and 300 µM Ca^2+^ (+Ca^2+^). Arrows indicate the addition of 20 µM Ca^2+^ (**D**) and 3 mM Trisma base (TB) or 5.8 mM HCl to reach the pH of media of 7.6 and 6.8, respectively €(**E**). Representative traces of one experiment of three that are similar are presented. The points of the curves are the means of three technical replicates.

The only reason for the difference in the matrix pH in S- and KCl-/NaCl-/LiCl-based media is the different mode of operation of the K^+^(Na^+^, Li^+^)/H^+^ exchanger (Figure 13A). In ChCl-BM, the exchanger works in the same mode, but the acidification of the matrix is prevented by the competing process of alkalinization via the Ch^+^/H^+^ exchanger. One can assume that the activation of the K^+^/H^+^ exchanger in S-BM leads to the acidification of the matrix which, in turn, inhibits the accumulation of P_i_ necessary for PTP induction. By contrast, in the presence of cations, the influx of H^+^ is either suppressed or compensated by its outflow. The data presented in Figure 13 support this assumption. The addition of 1 mg of the mitochondrial protein to S-BM led to a fast increase in the K^+^ concentration in the incubation medium, by 47.42 ± 1.61 μM, which corresponds to 25.7% of the total alamethicin-releasable pool of 184.3 ± 3.7 μM (Figure 13B,C). In NaCl-BM, the release of K^+^ was considerably suppressed and reached 30.37 ± 1.48 μM or ~16.5% of the total matrix content. The suppression of the initial release of K^+^ was associated with a faster Ca^2+^-dependent one (Figure 13B). We suggested that the bypass of the K^+^ efflux through the K^+^/H^+^ exchanger using the K^+^ ionophore valinomycin should prevent the acidification of the matrix in S-BM and increase the sensitivity of mitochondria to Ca^2+^. The results presented in Figure 13D,E support this suggestion. Valinomycin (50 ng/mL) strongly prevented the acidification of the matrix in S-BM (Figure 13E) and accelerated Ca^2+^-dependent swelling (Figure 13D), which was sensitive to n-ethylmaleimide (NEM), an inhibitor of the P_i_ carrier (PiC). Thus, monovalent cations regulate the PTP opening by modulating the activity of the K^+^/H^+^ exchanger which, in turn, regulates the matrix pH and hence the P_i_ transport into the matrix.

## 3. Discussion

In in vivo systems, ischemia/reperfusion causes PTP-dependent cell death [1,2]. Activators of K^+^ channels suppress PTP-dependent mitochondrial dysfunction and cell death [3,4,5,6,7,8,9,10]. Vice versa, the inhibitors of K^+^ channels, or their knockout, stimulate both mitochondrial dysfunction and cell death [6,7,8,9,10]. One can expect that the modulation of K^+^ transport should affect PTP opening in isolated mitochondria in the same way. Therefore, the idea of this work was to examine how the presence of K^+^ in the incubation medium affects the susceptibility of mitochondria to PTP opening, to compare the effect with the effects of other monovalent cations, and elucidate mechanisms underlying the effects revealed. 

We expected that the presence of K^+^ in the incubation media would protect isolated mitochondria from the Ca^2+^-dependent PTP opening, while other cations, namely Na^+^, Li^+^, and Ch^+^ would not. Against our expectations, the opening of the large (600 < P < 3350 Da) pore (PTP) was dramatically accelerated, and Ca^2+^-retention capacity was reduced in all cation-containing media tested in comparison with the cation-free S-BM (see Figure 2, Figure 4, Figure 5, Figure 6, Figure 7, Figure 8, Figure 9 and Figure 10). 

There are several possible reasons for the stimulation of PTP opening in the presence of salts. First, the ionic strength of the medium affects the interaction of charged groups of membrane phospholipids and proteins of the PTP complex, facilitating the pore opening. Second, the influx of cations or a chloride anion into the matrix triggers PTP opening via any mechanism. Third, salts affect PTP regulators independently of the cation/Cl^−^ influx into mitochondria. 

Experimental verification of the first possibility showed that the role of ionic strength in PTP regulation is very unlikely (see Figure 3). Indeed, the effect of different salt-based media of the same ionic strength on PTP opening was not the same. Moreover, the increase in the ionic strength from 15 (S-BM) to 19–20 mM (by the addition of KCl and ChCl) reduced the difference in the time of mitochondrial swelling by half. This implies too sharp a change in the intensity of ionic interactions upon a slight variation in the ionic strength.

Then, we examined the role of the electrophoretic influx of cations in the triggering of PTP opening. There are several established and hypothetical ways for the electrophoretic entry of cations into the mitochondrial matrix: selective K^+^ channels [71,94], the PTP being in a low-conductance state [28,74,75,76,77,78], and RR-sensitive non-selective cation channels [79]. 

The K^+^ channels are the most well-studied method for cations entering into the mitochondrial matrix [71,94]. However, they are quite selective for K^+^ and are poorer transporters for other cations [81]. The comparison of the effect of specific inhibitors of K^+^ channels (mK_ATP_, BK_Ca_, Kv_1.3_, and, to a lesser extent, Kv_1.1_, Kv_1.2_, Kv_1.4_, and Kv_1.7_) on PTP opening in K^+^-, Na^+^- and sucrose-containing media demonstrated that it is weak and unspecific (see Figure 4, Supplementary Figure S1). Hence, the acceleration of PTP opening by the electrophoretic influx of K^+^/Na^+^ through the selective K^+^ channels is negligible.

In several early studies, the PTP induction by Ca^2+^/P_i_ included two phases: the phase of PTP permeability to H^+^ and cations followed by the phase of permeability to sucrose and mannitol [75,95,96,97]. Recently, we observed a stepwise increase in PTP size (states of conductance) during the prolonged incubation of mitochondria. CsA slowed down and smoothed out the transitions between states [74]. However, in the present study, we did not find any signs of a preferential influx of cations through the PTP in the low-conductance state. Indeed, in S-BM, permeability to both sucrose (swelling) and H^+^ (ΔΨ_m_ loss) occurred simultaneously (Figure 2A,C) and much later than the swelling and ΔΨ_m_ loss in cation-containing media. Moreover, the efficiency of the protection of the mitochondria by CsA was almost independent of the concentration of cations in the incubation media (see Figure 3). This is contrary to what could be expected if the passage of cations through the small pore triggered the opening of the high-conductance PTP. Hence, presumably, cations and CyPD additively contribute to PTP opening.

One more way for the electrophoretic entry of cations into the matrix of energized mitochondria to trigger PTP opening is the hypothetical RR- and Mg^2+^-sensitive non-selective cation channel [79]. Though the channel is thought to be inhibited by submicromolar Mg^2+^ from both sides of the IMM, inhibition could be abolished by Ca^2+^ accumulation. However, the fact that RR added after Ca^2+^ accumulation strongly suppressed PTP opening, both in cation- and S-BM (Figure 6), shows that the protective effect is rather due to the inhibition of Ca^2+^ cycling across the IMM via MCU and TMBIM5 [83,91]. Accordingly, the bypass of the MCU with the Ca^2+^/Mg^2+^ ionophore A23187 canceled the protective effect of RR (see Figure 7). Moreover, the difference in the rate of PTP opening between cation- and S-containing media persisted in mitochondria de-energized by electron transport chain inhibitors and FCCP (see Figure 7). Thus, the hypothetic non-selective cation channel does not contribute to PTP being triggered by monovalent cations.

The simultaneous activation of the Cl^−^ and K^+^ influx into the mitochondria via the unidentified IMAC and any K^+^ transporter, respectively, strongly accelerates the mitochondrial swelling [98,99]. Therefore, we explored the effect of the potent IMAC inhibitors propranolol and Ro5-4864 [80] on PTP opening in different incubation media (Figure 8, Supplementary Material Figure S2). We found that the inhibitors are either ineffective or rather stimulatory in relation to PTP opening in all media, including S-BM. These data show that the entry of anions into the matrix via IMAC is dispensable for the stimulation of PTP opening in cation-based media. The activation of swelling may reflect the unspecific effects of the inhibitors on the system of volume regulation.

One more possible mechanism of stimulation of PTP opening by cations of the medium involves the activity of some cation exchangers. Indeed, in de-energized liver mitochondria, the activation of the K^+^/H^+^ exchanger inhibited the phenylarsine oxide-induced PTP via the acidification of the matrix [85]. Therefore, we evaluated the role of the K^+^ (Na^+^, Li^+^)/H^+^ [81] and Ch^+^/H^+^ exchanger CTL1/SLC44a1 [82] in the stimulation of the PTP by cations in energized mitochondria (Figure 9). The potent inhibitor of both exchangers of quinidine [82,88,89] affected PTP opening to a minimum extent in all cation-containing media tested. This refutes the importance of the activity of K^+^(Na^+^, Li^+^)/H^+^ and Ch^+^/H^+^ exchangers for the stimulation of PTP by cations. 

The data presented in Figure 6 and Figure 7 demonstrate that the Ca^2+^/H^+^ exchanger can be a potent inhibitor of PTP opening, at least when MCU is blocked. Hence, the rate of Ca^2+^ extrusion should inversely correlate with the rate of PTP opening. We hypothesized that cations in the media suppress the Ca^2+^ pumping out and accelerate the pore opening. However, this assumption has not received experimental confirmation (Figure 10), since the highest rates of Ca^2+^ extrusion were observed in the salt-containing media. Thus, salts sensitize mitochondria to PTP opening independently of the effect on the Ca^2+^/H^+^ exchange. Moreover, the effect of the external pH on PTP opening also did not confirm the role of cation exchangers in the PTP stimulation in salt-containing media (Figure 11).

In addition, liver mitochondria are capable of exchanging internal Ca^2+^ for external Na^+^ or Li^+^, though at a slow rate [100]. Being an important element of the maintenance of Ca^2+^ homeostasis in heart and brain mitochondria, the Ca^2+^/Na^+^ exchanger SLC8b1/NCLX is absent or scarce in liver mitochondria [84]. Therefore, the lack of the effect of its selective inhibitor CGP-37157 on PTP opening in liver mitochondria (Supplementary Figure S3) may be due rather to the absence of the carrier than its insignificant role in PTP regulation. 

Though the effect of the external pH on PTP opening did not confirm the role of cation exchangers in the PTP stimulation, it suggested that PTP may be triggered by exchangers of anions (Figure 11) [83,90,91,92]. At acidic external pH, the symport of P_i_ and glutamate with H^+^ via PiC and glutamate carriers Slc25a18/22, respectively, should rapidly acidify the matrix [92]. However, whether the low matrix pH suppresses or activates the PTP opening is not completely clear. On the one hand, the protonation of critical histidines of the PTP complex can inhibit PTP opening in certain conditions [93]. On the other hand, the stability of Ca^2+^-P_i_ complexes is decreased by acidic pH, which can raise the concentration of free Ca^2+^ in the matrix [30]. The measurements of the matrix pH in different incubation media revealed a great difference between S-BM and salt-based media in the first ~20 min of incubation (Figure 12, Supplementary Material Figure S4). Thus, the low matrix pH inhibits the PTP opening in accordance with the data of Nicoli and co-workers [93] and the presence of salts in the media precludes rapid matrix acidification. As follows from the scheme shown in Figure 13A, the only possible reason for the difference in the matrix pH in S- and KCl-, NaCl-, or LiCl-based media is the different mode of operation of the K^+^(Na^+^, Li^+^)/H^+^ exchanger. It should be activated in S-BM and suppressed (or operate in a bidirectional mode) in the presence of salts. In ChCl-BM, the exchanger presumably works in the same mode as in S-BM, but the acidification of the matrix is prevented by a competing process of alkalinization via the Ch^+^/H^+^ exchanger. The data obtained supports this assumption (Figure 13). Valinomycin, which causes a K^+^/H^+^ exchanger-independent release of matrix K^+^ in S-BM, both strongly suppressed matrix acidification and activated PTP opening. Moreover, in salt-based media, the release of matrix K^+^ was considerably diminished in comparison with S-BM. Hence, the K^+^/H^+^ exchanger plays a pivotal role in the PTP regulation but not as a passage for cation entry but as a regulator of matrix pH. 

The mechanism of PTP inhibition via an acidic matrix pH is presumably connected with the suppression of P_i_ transport. It was earlier established that the accumulation of P_i_ by energized mitochondria stimulates PTP opening [92]. The acidification of the matrix by the K^+^/H^+^ exchanger should inhibit the inward P_i_ transport via P_i_/OH^-^ antiport mediated by PiC. As NEM, an inhibitor of PiC, re-established the PTP inhibition abrogated by valinomycin in S-BM (Figure 13), this suggestion must be correct.

The data obtained reveal the role of monovalent cations in PTP regulation in vitro: suppression of the K^+^/H^+^-exchanger-mediated matrix acidification followed by the activation of inward P_i_ transport facilitating the PTP opening. Moreover, the data help to explain the protective effect of pre-conditioning caused by K^+^ channel activators in vivo: the K^+^/H^+^ exchanger-independent entry of K^+^ should activate K^+^/H^+^-exchanger-mediated matrix acidification and inhibit P_i_ accumulation and PTP opening. To conclude, the data obtained imply the great importance of the triad K^+^ channels/K^+^/H^+^-exchanger/PiC in the regulation of PTP in vivo.

## 4. Materials and Methods

### 4.1. Materials

Bovine serum albumin (BSA) (A2153), carbonyl cyanide p-(trifluoromethoxy)phenylhydrazone (FCCP) (C2920), Cyclosporin A (30024), digitonin (D141), 4-(2-hydroxyethyl)piperazine1-ethanesulfonic acid (HEPES) (H3375), rhodamine 123 (R8004), rotenone (R8875), sucrose (S7903), succinate (S3674), SF-6847/Tyrphostin A9 (T182), Trizma Base (93352), glybenclamide (G0639), psora-4 (P9872), quinidine (Q3625), iberiotoxin (recombinant from *Mesobuthus tamulus* (I5904)), quinine (145904), pinacidil monohydrate (P154), diazoxide (D9035), cgp-37157 (220005), and superoxide dismutase from bovine erythrocytes (S7571) were purchased from the Sigma-Aldrich Corporation (St. Louis, MO, USA). Ethylene glycol-bis(2-aminoethylether)-N,N,N0,N0-tetraacetic acid (EGTA) (A0878,0025) was obtained from PanReac AppliChem ITW Reagents (Darmstadt, Germany). Tetramethylrhodamine, methyl ester (TMRM), Calcein-AM, BCECF-AM (2’,7’-Bis-(2-Carboxyethyl)-5-(and-6)-Carboxyfluorescein, Acetoxymethyl Ester, (B1170)), Fluo-5F-AM (F14222) were obtained from Thermofisher (Waltham, MA, USA). Coomassie (Bradford) Protein Quantitative Assay Kit was obtained from Wuhan Servicebio Technology Co. (Wuhan, Hubei, China). Other chemicals were of analytical grade and were purchased from local suppliers. 

### 4.2. Isolation of Mitochondria from Rat Liver

All manipulations with animals before the isolation of organs were performed in accordance with the Helsinki Declaration of 1975 (revised in 1983), national requirements for the care and use of laboratory animals, and protocol 9/2020 of 17 February 2020 approved by the Commission on Biological Safety and Bioethics at the ITEB RAS. Adult male Wistar rats were decapitated after anesthesia with CO_2_. Rat liver mitochondria were isolated by a standard differential centrifugation procedure [101]. The homogenization medium contained 220 mM mannitol, 70 mM sucrose, 10 mM HEPES (pH adjusted to 7.4 with Trizma Base), 1 mM EGTA, and 0.05% BSA. The mitochondrial pellet was washed three times with EGTA- and BSA-free medium. Final pellets were resuspended in this medium to yield ~70 mg protein/mL. Mitochondrial protein was assayed by the Bradford method using BSA as a standard [102].

### 4.3. Incubation Media

Measurements were performed at 37 °C in isotonic (~300 mOsm) media, which contained 20 mM sucrose, 10 mM HEPES (pH adjusted to 7.3 with Trizma Base), 2 mM H_3_PO_4_, 2 mM MgCl_2_, and, as stated, either 5 mM succinate plus 2.5 µM rotenone or 5 mM glutamate plus 5 mM malate. The osmolarity of the solutions was adjusted to isotonic values with either 120 mM KCl (KCl-based medium, KCl-BM) or 120 mM NaCl (NaCl-based medium, NaCl-BM), or 120 mM choline chloride (ChCl-based medium, ChCl-BM), or 120 mM LiCl (LiCl-based medium, LiCl-BM), or 260 mM sucrose (sucrose-based medium, S-BM).

Media containing different concentrations of salts were prepared using a basic sucrose/HEPES/H_3_PO_4_/MgCl_2_ medium with the addition of the indicated concentrations of salts and necessary amounts of sucrose to establish osmolarity. 

In several experiments, media of different pH were applied. For this, the pH value of stock media was adjusted to 7.6 with Trizma Base. Just before the beginning of the recording, 1.05 and 2.35 µL of 400 mM HCl were added to the wells of 96-well plates with 100 µL of incubation media in order to shift the pH to 7.3 and 6.8, respectively.

### 4.4. Induction and Registration of PTP Opening

The induction of PTP opening occurs due to mitochondrial overload with Ca^2+^. There are three experimental approaches to induce Ca^2+^ overload: a gradual infusion of Ca^2+^ solution to mitochondrial suspension [103], the addition of Ca^2+^ in pulse(s) giving low final concentrations followed by prolonged incubation [55,56], and the addition of Ca^2+^ in pulse(s) giving high final concentrations followed by short incubation [60]. The first approach seems to be the most physiological but least convenient for high-throughput analysis. The last one operates with Ca^2+^ concentrations that far exceed those observed in any known physiological and pathophysiological states and can overcome any endogenous mitochondrial protective mechanism. Therefore, we applied a compromise experimental model: Ca^2+^ in concentrations close to the highest local concentrations near MAM-contacts (contacts of membranes of mitochondria and endoplasmic reticulum) [104] and a physiologically relevant incubation time (~1 h or ~2 h in S-BM) [56]. The opening of PTP in isolated mitochondria was registered as EGTA- and CsA-sensitive high-amplitude swelling, the dissipation of ΔΨ_m_, and the release of preliminarily accumulated Ca^2+^.

### 4.5. Recording of the Mitochondrial Swelling

Swelling is a dynamic process, which can be partly reversible in salt-based media in the presence of relatively low Ca^2+^ concentrations [105]. The susceptibility of mitochondria to Ca^2+^-dependent PTP opening was determined by two parameters: the half-maximum swelling time (HMST), which reflects the time when PTP opening occurs in half of the mitochondrial population [56]; and the integrated amplitude of absorbance (IA), which indicates the overall resistance of mitochondria. Mitochondrial swelling was recorded as a decrease in *A*_540_ in mitochondrial suspensions using a 96-well plate reader Infinite 200 Tecan (Groedig, Austria). Other details are given in the figures and figure legends.

### 4.6. Measurements of ΔΨm in Isolated Mitochondria

Additionally, ΔΨ_m_ across the IMM was measured using the ΔΨ_m_-sensitive fluorescent dye rhodamine 123 and a plate reader Infinite 200, Tecan (Groedig, Austria). Incubation media contained respiratory substrates, 330 nM rhodamine 123, and, where indicated, 1 mM EGTA and 1 µM CsA. Other additions (Ca^2+^ and 500 nM FCCP) were made in the course of recordings. In order to calibrate the fluorescent signal, each experimental series contained samples with a cocktail of respiratory inhibitors and ionophores, which disrupt ionic gradients across the IMM (500 nM FCCP, antimycin A (2.5 μg/mL), and valinomycin (25 ng/mL)) [75,106]. In addition, ΔΨ_m_ was calculated using the Nernst equation, assuming that the matrix volume is equal to 1 µL/mg protein (liver mitochondria) and that the fluorescence of rhodamine 123 is directly proportional to the concentration in solution and is totally quenched upon accumulation in the mitochondria.

### 4.7. Ca^2+^-Retention Capacity of Mitochondria

Mitochondrial Ca^2+^ uptake and release were recorded in a temperature-controlled electrode chamber using a Ca^2+^-electrode (Niko Analyt, Moscow, Russia) connected to the computerized recording system Record 4 (ITEB RAS, Puschino, Russia). The electrode was calibrated with known amounts of Ca^2+^ at the beginning of each experimental series. The CRC of the mitochondria was defined as the amount of Ca^2+^ taken up by the mitochondria in small pulses before the Ca^2+^ release. Other experimental details are given in figure legends.

### 4.8. Measurements of the Oxygen Consumption Rate

The mitochondrial respiration was measured using an Oroboros Oxygraph-2k device (Vein, Austria). Mitochondria (1 mg/mL) were incubated at 25 °C in incubation media supplemented with 5 mM glutamate and 5 mM malate. In order to assess state 3 (*V*_3_) and state 4 respiration rates (*V*_4_), 200–500 μM ADP was added to mitochondria respiring in the presence of substrates. The respiratory control coefficients were calculated as the ratio of respiration rates in state3/state4. The P/O ratio was calculated as the amount of completely phosphorylated ADP per number of oxygen atoms consumed.

### 4.9. Registration of the Release of Matrix K^+^ from Mitochondria

The release of mitochondrial K^+^ was recorded in a temperature-controlled electrode chamber using a K^+^-electrode (Niko Analyt, Moscow, Russia) connected to the computerized recording system Record 4 (ITEB RAS, Puschino, Russia). The electrode was calibrated with known amounts of KCl at the beginning of each experimental series. Other experimental details are given in figure legends.

### 4.10. Determination of Matrix pH

The matrix pH of liver mitochondria was estimated as described previously [107], with some modifications. Briefly, mitochondria (12 mg) were suspended in 1 mL of medium containing 220 mM mannitol, 70 mM sucrose, 10 mM HEPES (pH adjusted to 7.4 with Trizma Base), and 10 μg of BCECF-AM and incubated on ice in the dark for 30 min. The mitochondria were incubated at room temperature for an additional 30 min. Then, the mitochondria were sedimented at 14,000× *g* for 10 min and washed twice. The final pellet was suspended in 0.1 mL of the same medium without BCECF-AM and kept on ice until use. The fluorescence of BCECF trapped in the matrix was measured in a plate reader Infinite 200, Tecan (Ex 480 nm, Em 525 nm). A BCECF signal was calibrated either using a range of buffers of known pH (6.8–7.6) and equilibrating the matrix and external pH using alamethicin (40 µg/mg prot.) or by the addition of known amounts of Trizma-Base and HCl solutions to mitochondria incubated with 300 µM Ca^2+^.

### 4.11. Statistical Analysis

The data shown represent the means ± standard error of means (S.E.M.) or are the means of at least three experiments. Statistical probability (*p*) values were derived by the Student’s *t*-test.

## Data Availability

All data related to the manuscript can be found in the manuscript body, Supplementary Information file, or upon request.

## Supplementary Materials

The supporting information can be downloaded at: https://www.mdpi.com/article/10.3390/ijms24119237/s1.
