# Supplementary material for: Regulation of Mitochondrial Permeability Transition Pore Opening by Monovalent Cations in Liver Mitochondria
Source: Int J Mol Sci. 2023 May 25;24(11):9237. doi: 10.3390/ijms24119237 (PMC10252971; doi:10.3390/ijms24119237)
Supplement: Supplementary file 1 [file ijms-24-09237-s001.zip › ijms-2382365-supplementary.pdf]

Supplementary Materials

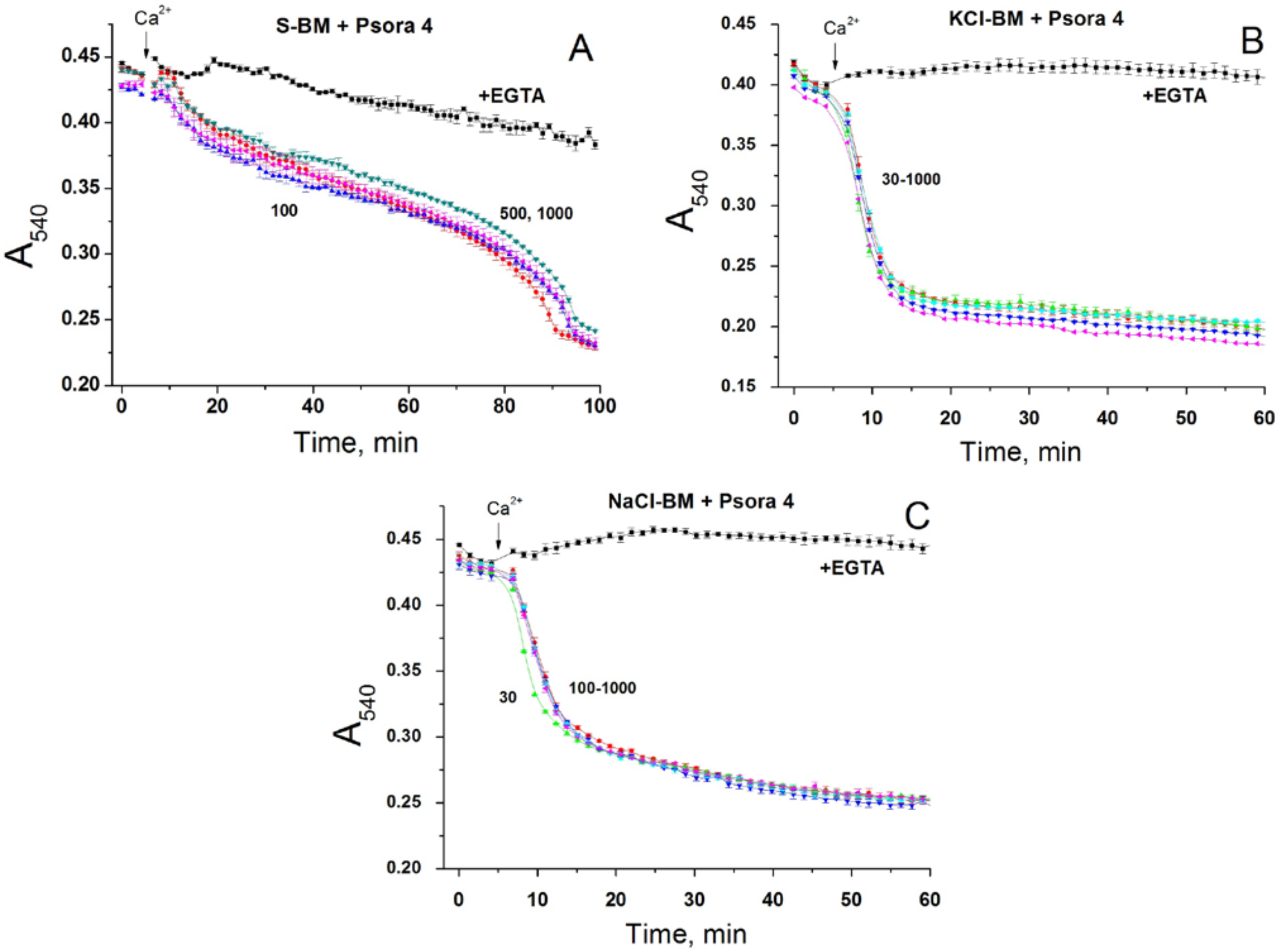

Figure S1. Effect of Psora 4 at high (30-1000 nM) concentrations on the PTP opening in mitochondria in different incubation media. Two min before measurements, RLM (0.75 mg protein/ml) were placed in the incubation media supplemented with 5 mM glutamate plus 5 mM malate (Trizma salts), 10 µM EGTA and indicated concentrations of Psora 4 (in nM). Red lines correspond to Ca^{2+}-dependent swelling in the absence of other additions. Green, blue, magenta, and cyan colors show the presence of 30, 100, 500, and 1000 nM psora 4, respectively. Arrows show the addition of 20 µM Ca^{2+}. Traces of two experiments of four similar are presented. Values on traces are means ± S.E.M. for three technical replicates.

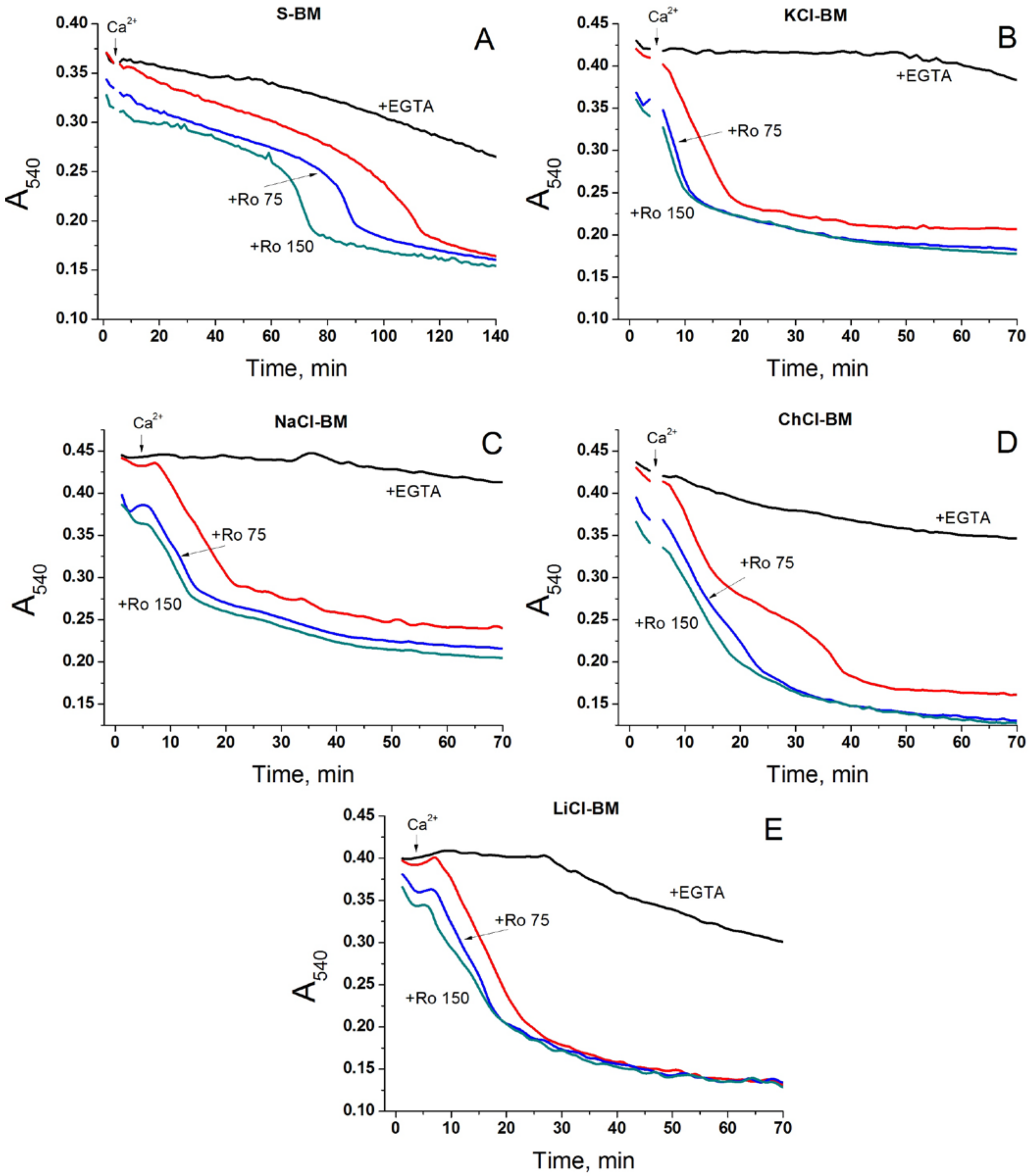

Figure S2. Effect of Ro5-4864 on the Ca^{2+}-dependent mitochondrial swelling in KCl-, NaCl-, ChCl-, LiCl-, and S-BM. Two min before measurements, RLM (0.75 mg protein/ml) were placed in the incubation media supplemented with 5 mM glutamate plus 5 mM malate (Trizma salts), 10 µM EGTA and, where indicated, 1 mM EGTA (+EGTA), 75 or 150 µM Ro5-4864. Five minutes after the start of recordings, 20 µM Ca^{2+} was added to the samples with standard (10 µM) EGTA. Red lines correspond to Ca^{2+}-dependent swelling in the absence of other additions. Incubation media are S-BM (A), KCl-BM (B), NaCl-BM (C), ChCl-BM (D), and LiCl-BM (E). Traces of one representative

experiment of three similar are presented. Values on traces are means for three technical replicates. Arrows show the addition of 20 μM Ca^{2+} (final concentration).

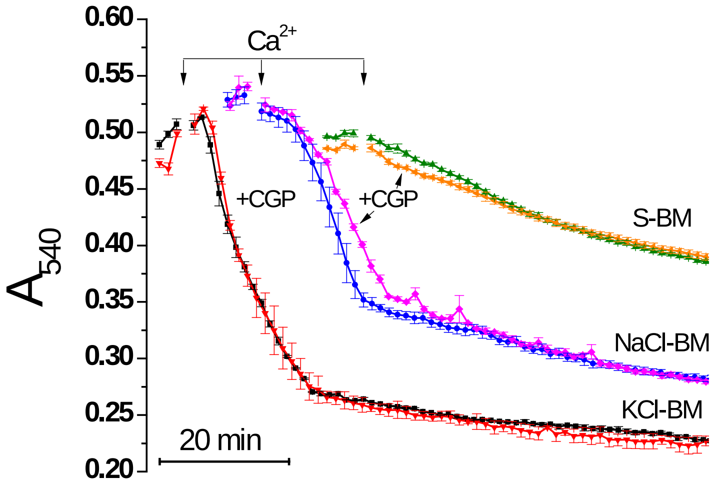

Figure S3. Effect of CGP-37157 on the Ca^{2+}-dependent mitochondrial swelling in KCl-, NaCl-, and S-BM. Incubation conditions and protocol were as in Fig. 3. Where shown, media contained 10 μM CGP-37157 (+CGP). Arrows indicate the addition of 20 μM Ca^{2+}. Traces were shifted by 10 (NaCl-BM) and 25 min (S-BM) for clarity. Traces of one representative experiment of three similar are presented. Values on traces are means ± S.E.M. for three technical replicates.

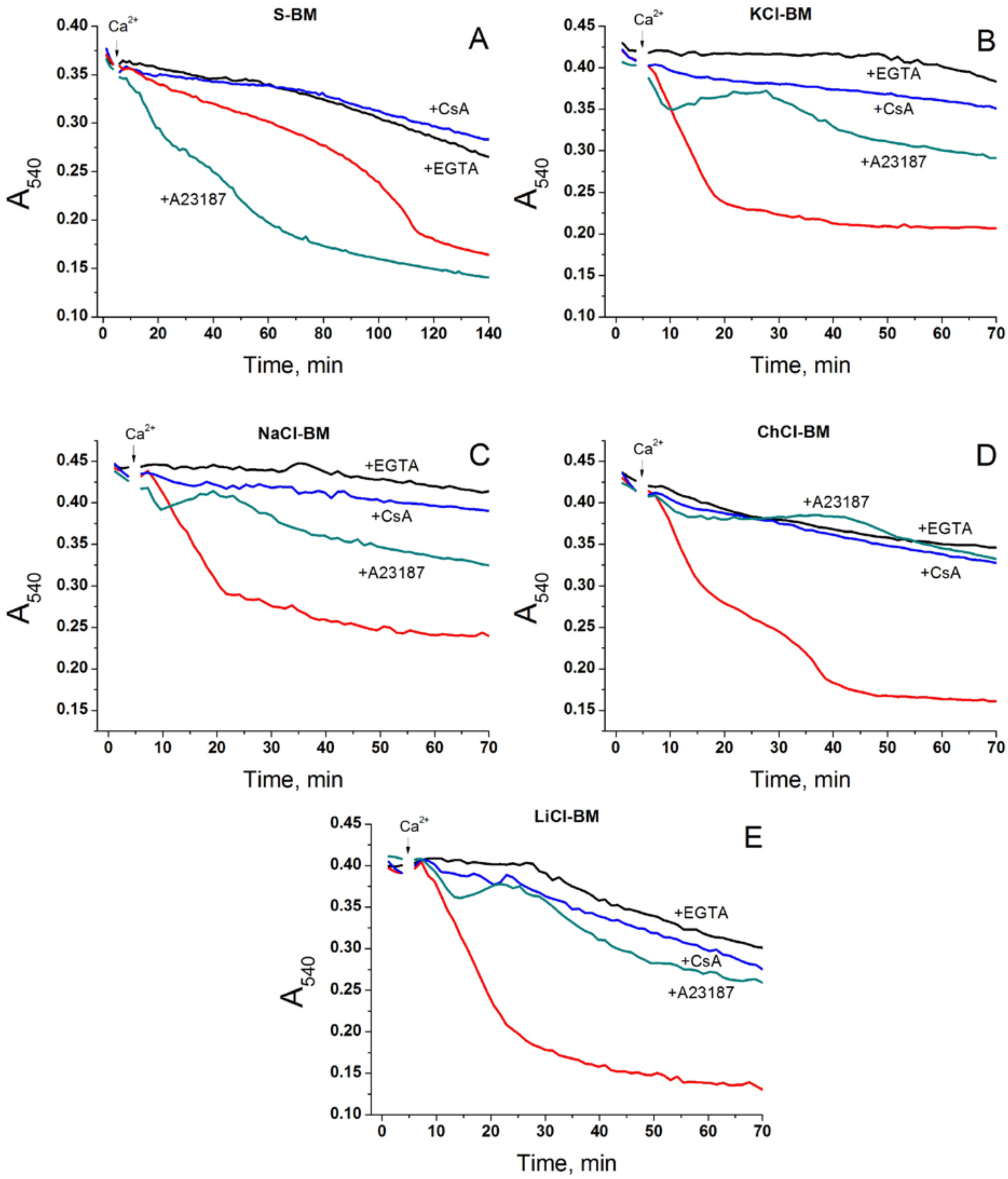

Figure S4. Effect of A23187 on the Ca^{2+}-dependent mitochondrial swelling in energized mitochondria incubated in S-, KCl-, NaCl-, ChCl-, and LiCl-BM. Two min before measurements, RLM (0.75 mg protein/ml) were placed in the incubation media supplemented with 5 mM glutamate plus 5 mM malate (Trizma salts), 10 µM EGTA and, where indicated, 1 mM EGTA (+EGTA), 1 µM CsA and 10 µM A23187. Five minutes after the start of recordings, 20 µM Ca^{2+} was added to the samples with standard (10 µM) EGTA concentrations. Red lines correspond to Ca^{2+}-dependent swelling in the absence of other additions. Incubation media are S-BM (A), KCl-BM (B), NaCl-BM (C), ChCl-BM (D), and LiCl-BM (E). Traces of one representative experiment of three similar are presented. Values on traces are means for three technical replicates. Arrows show the addition of 20 µM Ca^{2+} (final concentration).
